# Differential Expression of *VvLOXA* Diversifies C6 Volatile Profiles in Some *Vitis vinifera* Table Grape Cultivars

**DOI:** 10.3390/ijms18122705

**Published:** 2017-12-20

**Authors:** Xu Qian, Lei Sun, Xiao-Qing Xu, Bao-Qing Zhu, Hai-Ying Xu

**Affiliations:** 1 Department of Food Science and Engineering, College of Biological Sciences and Technology, Beijing Forest University, Beijing 100083, China; qianxu@cau.edu.cn (X.Q.); xuxiaoqing@outlook.com (X.-Q.X.); 2 Beijing Academy of Forestry and Pomology Sciences, Beijing 100093, China; sunlei.bjfu@gmail.com

**Keywords:** *Vitis vinifera* grapes, C6 volatiles, gas chromatography-mass spectrometry (GC-MS), *VvLOXA* expression, lipoxygenase-hydroperoxide lyase (LOX-HPL) pathway, multivariate analysis

## Abstract

C6 volatiles are synthesized through lipoxygenase-hydroperoxide lyase (LOX-HPL) pathway and these volatiles play important roles in the aromatic quality of grape berries. This study investigated the evolution of both C6 volatiles and the key genes in the LOX-HPL pathway in different table grape cultivars during the berry development period, and further assessed the correlation between the accumulation of C6 volatiles and the expression of these genes in these cultivars. Results showed that hexanal, (*E*)-2-hexenal, (*E*)-2-hexen-1-ol and (*Z*)-3-hexen-1-ol were found to be the dominant C6 volatiles in these ripened grape cultivars under two consecutive vintages, and their flavor notes were incorporated in the overall aroma of these cultivars. The cultivar “Xiangfei” showed the most abundant level of C6 aldehydes and C6 acid, whereas the cultivar “Tamina” and “Moldova” possessed the highest C6 alcohol content. The “Muscat of Alexandria” cultivar was found to contain the highest level of C6 esters. C6 volatiles were grouped into three evolutionary patterns in these cultivars during berry development, and their evolution was consistent with the evolution of the LOX-HPL pathway genes’ expression. Pearson’s correlation analysis indicated that the LOX-HPL-pathway-related genes were correlated to the accumulation of C6 volatiles in these cultivars, and *VvLOXA* appeared to be an important gene that regulated the synthesis of all C6 volatiles.

## 1. Introduction

China has become one of the world’s leading grape-producing countries, with an annual grape yield of 12.6 million tons in 2014 [1]. Eighty percent of its grapes are table grapes that are normally eaten fresh by customers. Additionally, 15% of the total grape yield is used to produce wine, while grape raisins are made from 5% of the total grape output [2]. Regarding table grapes, their berry size, appearance, texture, and sensory attributes play an essential role in determining the market value and acceptance of customers. It has been confirmed that these nutritional parameters of table grapes are affected by grape genetic backgrounds [3,4,5], cultivation conditions [6,7,8], post-harvesting operations [9,10], and packaging treatments [11,12].

The overall aroma of fruits has been confirmed to be determined by the volatile composition [13,14,15,16]. It has been reported that grape berries contain numerous volatile compounds. Among these volatile compounds, C6 volatile compounds exist as one of the major volatile compounds in grape berries and other fruits [17,18,19,20]. These volatiles have been reported to exhibit vegetable, fresh, green, fruity, almond, apple, or minty scents [21,22].

Biologically, the biosynthesis of C6 volatile compounds in fruits results from oxylipin metabolism under the activity of lipoxygenase (LOX), hydroperoxide lyase (HPL), alcohol dehydrogenase (ADH), and alcohol acetyltransferase (AAT) in the LOX/HPL pathway [23]. The composition and distribution of C6 volatile compounds in grape berries is determined by grape germplasm, cultivar, climate, soil, vintage, and cultivation management [14,17,24,25,26,27]. For instance, it has been reported that alteration of the microclimate, such as light exposure duration and water conditions, could inhibit or stimulate the expression of the LOX/HPL-pathway-related genes, which further results in alteration of the activity of C6 volatiles’ synthesis-related enzymes. As a result, C6 volatiles’ accumulation in grape berries could be significantly altered [28,29,30]. Such investigations have been well studied in wine-making grape cultivars. However, few studies were focused on table grape cultivars, to our best knowledge. Therefore, this study was designed to investigate C6 volatiles’ accumulation and related gene expression in table grape berries, with the aim of improving table grape berries’ quality. To this end, we selected seven cultivars of *Vitis vinifera* table grape berries, including “Xiangfei”, “Moldova”, “Tamina”, “Italia”, “Zaomeiguixiang”, “Muscat of Alexandria”, and “Christmas Rose” in two consecutive vintages (2013 and 2014). These cultivars are mainly cultivated as table grapes in China, and they possess different sensory features. In each vintage, the C6 volatiles composition of each cultivar from veraison to harvest was studied using solid-phase microextraction (SPME)–gas chromatography/mass spectrometry (GC/MS). More importantly, unlike other studies [31], the concentration of the C6 volatile compounds in these cultivars was absolutely quantified with their standard in the present study. Meanwhile, the expression of the LOX-HPL-pathway-related genes in these cultivars (except “Moldova” and “Italia”) in the 2014 vintage was assessed using the real-time qPCR technique. The findings from this study could elucidate the contribution of the C6 volatile composition to the aromatic features of table grape berries, and further establish the correlation of the C6 volatiles’ accumulation with the critical genes in table grapes during berry development stages.

## 2. Results and Discussion

### 2.1. C6 Volatile Level, Odor Contribution, and Cultivar and Vintage Effect

**Figure 1:**
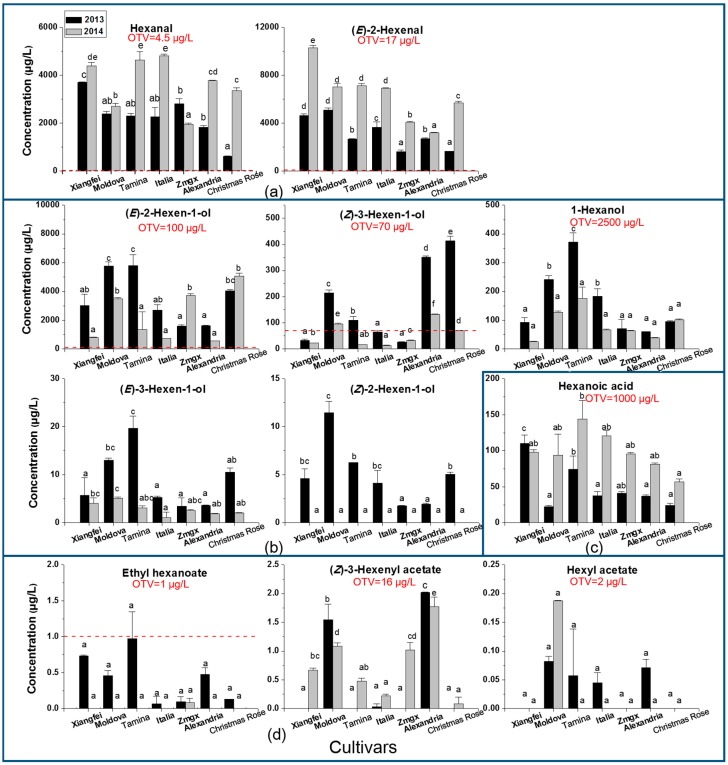
Concentrations of volatile compounds and their odor threshold values in different table grape cultivars at harvest. (**a**) C6 aldehydes; (**b**) C6 alcohols; (**c**) C6 acid; and (**d**) C6 esters. Different letters mean significant differences according to the Duncan test (*p* < 0.05) in individual vintages. “Zmgx” represents the “Zaomeiguixiang” cultivar; “Alexandria” represents the “Muscat of Alexandria” cultivar; OTV is the odor threshold value.

C6 volatile compounds are accumulated in grape berries along with the berry development stages, and their level in ripened grape berries determines the overall aroma [18,24,26]. In the present study, a total of 11 C6 volatiles were detected in these table grapes at harvest. Regarding their chemical feature, these C6 volatiles included two aldehydes, five alcohols, three esters, and one acid under these two vintages (Figure 1). The highest concentration of the total C6 volatile compounds was found in the “Xiangfei” cultivar at the harvest under both vintages, whereas the ripen “Christmas Rose” cultivar exhibited the least content, especially in 2013.

Regarding the C6 aldehydes, hexanal and (*E*)-2-hexenal were found in all table grapes at harvest, and these two aldehydes appeared to be the dominant C6 volatiles. The concentration of these two aldehydes in these ripenedgrapes ranged between 1–5 mg/L and 2–10 mg/L, respectively, which was significantly higher than their odor threshold (4.5 µg/L and 17 µg/L, [32]). This indicated that their greenish and fruity flavor notes could significantly affect the overall aroma of these table grapes [21]. It has been reported that C6 aldehydes also played important roles in affecting the aromatic feature of other fruits due to their high concentration [19,20]. Among these ripenedtable grape samples, the “Xiangfei” cultivar was found to exhibit the highest level of these C6 aldehydes in both the 2013 and 2014 vintages, whereas the lowest level of these aldehydes were observed in the “Christmas Rose” cultivar at the ripening stage in 2013 (Figure 1a).

In terms of the C6 alcohols, all the table grape cultivars contained these five individual C6 alcohols in both vintages (except for (*Z*)-2-hexen-1-ol in 2014) at harvest (Figure 1b). The “Moldova” cultivar exhibited the highest level of total C6 alcohols, whereas the lowest was found in the “Muscat of Alexandria” cultivar at harvest in both vintages. Meanwhile, the 2013 ripenedgrape cultivars possessed a higher concentration of the total C6 alcohols than those harvested in 2014. (*E*)-2-hexen-1-ol appeared to be the dominant C6 alcohol in these grapes, which was different to some wine-making grapes [18,26]. Additionally, these table grape cultivars at the harvest contained a higher content of (*E*)-2-hexen-1-ol than (*Z*)-3-hexen-1-ol. Similar reports were also observed in other table grape cultivars [17,31]. Among these table grape cultivars, the “Tamina” and “Moldova” cultivars in the 2013 vintage showed the highest concentration of (*E*)-2-hexen-1-ol, 1-hexanol and (*E*)-3-hexen-1-ol, whereas (*Z*)-3-hexen-1-ol was more present in the “Muscat of Alexandria” and “Christmas Rose” cultivars. C6 alcohols have been reported to have herbaceous, grassy, green, and leaf-like scents [33,34]. In these grape cultivars, 1-hexanol and (*E*)-3-hexen-1-ol did not have a concentration higher than their odor threshold, indicating that these volatiles could provide a limited contribution to the overall aroma of these grapes. However, (*E*)-2-hexen-1-ol exhibited a concentration higher than the threshold in these table grapes, demonstrating that its flavor notes could be incorporated in the aromatic features of these grapes. It should be noted that the flavor contribution of (*Z*)-3-hexen-1-ol varied in these grapes. For example, its concentration was higher than its odor threshold in the “Moldova”, “Muscat of Alexandria”, and “Christmas Rose” cultivars at both vintages. However, the “Xiangfei” and “Zaomeiguixiang” cultivars showed a low concentration of (*Z*)-3-hexen-1-ol at harvest. Its scent notes were only emphasized in the “Tamina” cultivar in 2013.

C6 esters have been reported to be important volatiles that provide wine-making grapes (such as Merlot and Cabernet Sauvignon) and other fruits with varietal flavor notes [34,35]. Their flavor scents have been depicted as fruity, floral, and sweet in fruits [34,35]. In the present study, only ethyl hexanoate exhibited a concentration close to its odor threshold in the “Tamina” cultivars under the 2014 vintage (Figure 1d). (*Z*)-3-Hexenyl acetate and hexyl acetate appeared to be barely above their threshold in these cultivars.

Hexanoic acid was the only C6 acid found in these grape cultivars at harvest (Figure 1c). Compared to the C6 aldehydes and alcohols, this volatile exhibited a low concentration in these table grape cultivars (40 to 120 µg/L). Among these cultivars, the “Xiangfei” and “Tamina” cultivars showed the highest level of hexanoic acid, whereas the lowest level of this acid was found in the “Christmas Rose” cultivar at harvest. This C6 acid has been reported to possess sweaty, cheesy, and fatty flavor notes [36]. However, its scent could not significantly contribute to the overall aroma of these grape cultivars due to its high odor threshold [37].

A two-way ANOVA was carried out in this study to elucidate the effect of cultivar, vintage, and cultivar x vintage interaction on the C6 volatile composition in these table grape cultivars (Table 1). It was observed that the grape cultivar played a primary effect on the composition of these C6 volatile compounds, except in ethyl hexanoate, whereas the vintage exerted a significant effect on the accumulation of these volatiles except for hexyl acetate. These results were consistent with the previously published study [24,27]. Moreover, the cultivar × vintage interaction played a role in affecting the level of these volatile compounds, except for ethyl hexanoate and 1-hexanol. These results indicated that these table grape cultivars had significant differences in terms of the C6 volatile composition and distribution.

### 2.2. Evolution of C6 Volatiles in Grapes during Berry Development

**Figure 2:**
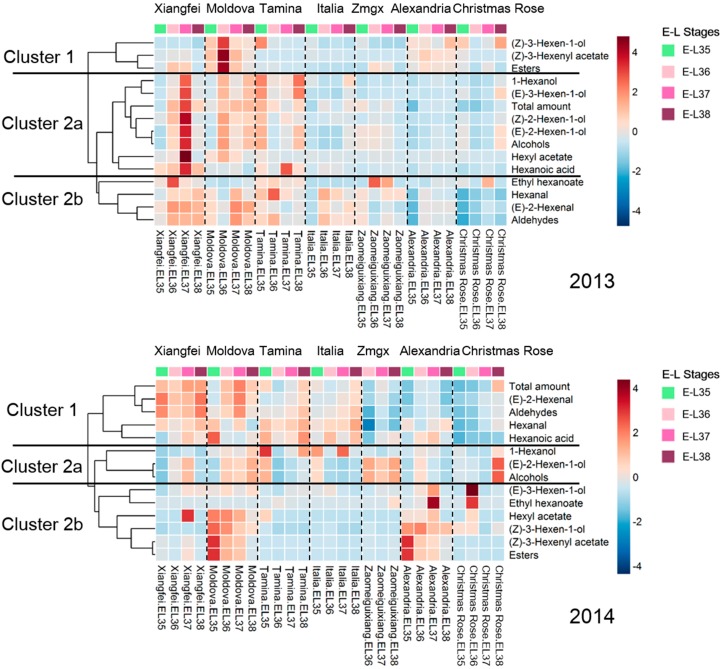
Heat maps of C6 volatiles in different table grape cultivars during berry development in 2013 and 2014. “Zmgx” represents the “Zaomeiguixiang” cultivar. “Alexandria” represents the “Muscat of Alexandria” cultivar.

These C6 volatiles were clustered into three groups (Cluster 1, 2a,b) regarding their evolution similarity in these table grape cultivars during the berry development stages under both vintages (Figure 2). All the C6 aldehydes in these cultivars exhibited a similar evolution pattern (Cluster 2b in 2013 and Cluster 1 in 2014). For example, the C6 aldehydes in the “Xiangfei” cultivar remained at a high level during the veraison stage, followed by a dramatic increase during the harvest in 2014. The C6 aldehydes increased in concentration in the “Moldova”, “Tamina”, “Italia”, and “Muscat of Alexandria” cultivars during the beginning stage of the veraison, followed by a concentration decrease before the harvest in the 2013 vintage. Regarding the individual C6 aldehydes, (*E*)-2-hexenal and hexanal showed an increase during berry maturation, followed by a decrease at the harvest in most of the cultivars in 2013. A similar evolution pattern of these aldehydes was also observed in Riesling and Cabernet Sauvignon grapes [24]. In 2014, these aldehydes did not decrease their concentration in “Tamina” and “Italia” before the harvest stage.

Most of the C6 alcohols were grouped into Cluster 2a in these table grape cultivars under these two vintages (Figure 2). The C6 alcohols in the “Xiangfei” cultivar exhibited increased levels during berry development, followed by a dramatic reduction at the harvest in 2013. In the 2014 vintage, only 1-hexanol, (*E*)-2-hexen-1-ol, and the total C6 alcohols showed a similar evolution pattern (Cluster 2a). It has been reported that 1-hexanol and (*E*)-2-hexen-1-ol showed an increased level and then a decrease pattern in the ‘Jingxiu’ table grape cultivar during berry development, whereas a continuous accumulation of 1-hexanol was found in the Riesling and Cabernet Sauvignon cultivars [24,31]. The C6 alcohols in the “Moldova”, “Tamina”, “Italia”, “Muscat of Alexandria”, and “Christmas Rose” cultivars continued to accumulate through the veraison to the harvest stage in the 2013 vintage. However, the C6 alcohol content showed a decreasing pattern in the “Tamina”, “Italia”, and “Muscat of Alexandria” cultivars during berry development under the 2014 vintage. The “Zaomeiguixiang” cultivar exhibited a continuous decrease in the C6 alcohols during the berry development period in 2013. However, its alcohol content was relatively stable in the 2014 vintage.

The C6 esters in these cultivars during the development were assembled in Clusters 1 and 2a in the 2013 vintage, whereas they were grouped into Cluster 2b under the 2014 vintage (Figure 2). It was observed that these esters rapidly accumulated at the early stage of development, followed by a decrease at the harvest stage in both vintages. Such a trend was more obvious in the “Moldova” and “Muscat of Alexandria” cultivars. Our observation was consistent with previous reports [18,24]. Regarding hexanoic acid (the only C6 acid detected in these cultivars), it showed an increase and then a decrease in its level during the maturation period in these cultivars, especially “Xiangfei” and “Tamina”, under the 2013 vintage. In 2014, a similar evolution pattern was also found in the “Xiangfei” and “Muscat of Alexandria” cultivars. It should be noted that an increase of hexanoic acid was observed in the “Tamina” cultivar before the harvest.

### 2.3. Transcript Level of Key LOX-HPL Pathway Genes during Development

C6 volatile compounds have been confirmed to be produced via the LOX-HPL pathway in grape berries during the berry development period, and it has been reported that lipoxygenase (LOX), hydroperoxide lyase (HPL), alcohol dehydrogenase (ADH), and alcohol acetyl transferase (AAT) are the key enzymes that regulate the biosynthesis of C6 volatiles [18,38,39,40]. In order to elucidate the effect of the LOX-HPL pathway key genes on the accumulation of C6 volatiles in these table grape cultivars, we further investigated the evolution of the transcript level of the genes in these cultivars under the 2014 vintage.

**Figure 3:**
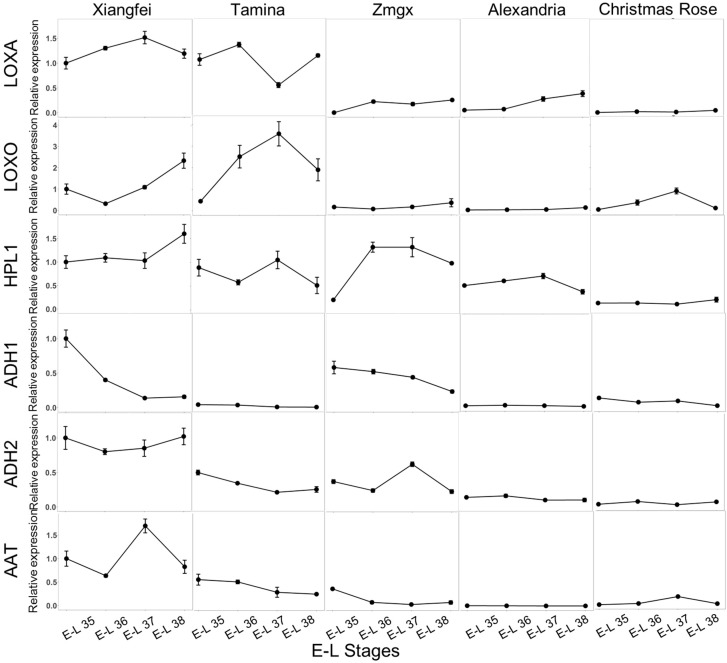
Transcript levels of key genes in LOX/HPL pathways in different table grape cultivars during berry development in 2014. GenBank accession numbers are as follows: *VvLOXA* (FJ858255), VvLOXO (FJ858257), *VvHPL1* (HM627632), *VvADH1* (AF194173), *VvADH2* (AF194174) and *VvAAT* (AAW22989). E-L 35 (early veraison), E-L 36 (mid-ripening stage), E-L 37 (end of veraison), and E-L 38 (harvest). “Zmgx” represents “Zaomeiguixiang” cultivar and “Alexandria” represents “Muscat of Alexandria” cultivar. Expression levels of each gene were expressed as a ratio relative to the E-L 35 stage of “Xiangfei” cultivar, which was set at 1.

Lipoxygenases (LOXs) have been reported to take charge of yielding PUFAhydroperoxides via catalysis of the oxygenation of polyunsaturated fatty acids (PUFAs) [38]. *VvLOXA* and *VvLOXO* have been considered representative of putative 13-LOXs [38]. In the present study, an increase in the expression of the *VvLOXA* was observed in these cultivars after veraison, and then its expression exhibited a decrease when the grape berries reached the harvest stage (Figure 3). Additionally, the expression level of the *VvLOXA* appeared to be higher in the “Xiangfei” and “Tamina” grape cultivars. It should be noted that *VvLOXA* exhibited much higher expression in these grape cultivars than *VvLOXO.* A similar observation was also reported in wine-making grape cultivars [27]. Therefore, *VvLOXA* was speculated to be the primary 13-LOX in these cultivars. The transcript level of *VvLOXO* was higher in the “Tamina” and “Xiangfei” cultivars. However, its expression pattern was totally different. For example, an increase and then a decrease in the expression of the *VvLOXO* was found in the “Tamina” cultivar during the maturation period, whereas the “Xiangfei” cultivar resulted in a decrease and then an increase in the *VvLOXO* expression. Additionally, the “Zaomeiguixiang”, “Muscat of Alexandria”, and “Christmas Rose” cultivars displayed a low expression level of these LOXs during the whole berry development period (Figure 3), which resulted in a low concentration of the C6 aldehydes in these grape cultivars (Figure 2).

Hydroperoxide lyases (HPLs) are the key enzymes that can cleave PUFAhydroperoxides into aldehydes and oxoacids [39]. The *VvHPL1* gene was expressed at a higher level in the “Xiangfei” and “Zaomeiguixiang” cultivars during development. However, its expression pattern in these cultivars appeared to be significantly different (Figure 3). For example, the expression of *VvHPL1* remained stable in the “Xiangfei” cultivar after veraison, and a further increase in the gene expression was observed when the grapes approached the harvest. However, this gene was rapidly expressed in the “Zaomeiguixiang” cultivar from the beginning of the veraison stage, and then a decrease in its expression level was found at the late stages of development. A similar evolution of this gene was reported in the Cabernet Sauvignon cultivar during berry development [39]. Regarding the other cultivars, the *VvHPL1* gene was expressed at a constant level, followed by a decrease at the harvest. The evolution of this gene expression in these cultivars was consistent with the evolution of the C6 aldehydes during berry development.

Alcohol dehydrogenases (ADHs) can convert alcohols into aldehydes and two key genes (*VvADH1* and *VvADH2*) have been confirmed to biosynthesize the ADHs in grape berries [40]. It has been reported that *VvADH2* played a primary role in facilitating berry ripening through enhancing its expression level at the late stage of berry development, whereas berry maturation resulted in a decrease in *VvADH1* expression [40]. In the present study, a higher transcript level on the *VvADH2* was found in the “Xiangfei” cultivar, and this gene expression exhibited a decrease after the veraison and then an increase at the harvest (Figure 3). However, the rest of the grape cultivars showed a constant decrease in the expression of the *VvADH2* throughout the berry development stages. Additionally, higher expression of *VvADH1* was observed in the “Xiangfei” and “Zaomeiguixiang” cultivars. The berry maturation process of these two cultivars resulted in a decrease in its expression. A previous study has reported that *VvADH1* expression was enhanced before veraison and then inhibited when wine-making grapes approached ripeness [26].

The reactions between alcohols and acetyl coenzyme A have been confirmed to be catalyzed by alcohol acetyltransferase (AAT), and such reactions could result in the formation of esters [18]. A high transcription level of *VvAAT* was found in the “Xiangfei” cultivar. During the berry development period, the expression of this gene exhibited a significant increase and then a dramatic decrease under the 2014 vintage. However, the transcription level of this gene was low in the other cultivars, and an expression decrease was found from the veraison to the harvest stage. This evolutionary pattern of *VvAAT* was not in accordance with that in Cabernet Sauvignon [27]. It should be noted that only the evolutionary pattern of the *VvAAT* in the “Xiangfei” cultivar matched the evolutionary pattern of the C6 esters (Figure 2).

### 2.4. Correlation of Genes and C6 Volatiles

To elucidate the relationship between these LOX-HPL pathway key genes and the C6 volatiles accumulated in these cultivars, a Pearson’s correlation analysis was carried out (Table 2). It was found that the *VvLOXA* expression was strongly positively correlated with the total C6 volatiles, total aldehydes, and total alcohols in these cultivars during berry development. This indicated that the differential expression of the *VvLOXA* played a primary role in diversifying the C6 volatile profiles in these cultivars. For example, a high level of the C6 volatiles in the “Xiangfei” and “Tamina” resulted mainly from the high expression of *VvLOXA* during the maturation period. The differential expression of *VvLOXA* in these cultivars might be attributed to the nucleotide sequence diversity in either the non-coding regulation region of the gene encoding a key enzyme or the promotor region of an unknown transcriptional factor that can activate the key gene expression [41,42,43]. However, a further study should be carried out to elucidate the regulation mechanisms. In addition, the accumulation of the C6 aldehydes and C6 acid was related to the expression of *VvADH2* and *VvHPL1* since a correlation was found among *VvADH2*, *VvHPL1*, hexanal, (*E*)-2-hexenal, and hexanoic acid. The content of hexyl acetate was closely correlated with the *VvAAT* expression.

## 3. Conclusions

In conclusion, the “Xiangfei” cultivar possessed the highest concentration of total C6 volatiles at harvest, whereas the lowest total C6 volatiles level was found in the “Christmas Rose” cultivar in both 2013 and 2014 vintages. Hexanal, (*E*)-2-hexenal, (*E*)-2-hexen-1-ol, and (*Z*)-3-hexen-1-ol appeared to be the dominant individual C6 volatiles that contributed their flavor notes to the overall aroma of these table grape cultivars. The “Xiangfei” cultivar exhibited the highest level on C6 aldehydes and C6 acid, whereas the highest level of C6 alcohols was found in the “Tamina” and “Moldova” cultivars. The “Muscat of Alexandria” cultivar possessed the highest level of esters at harvest. Regarding their evolution patterns, C6 volatiles in these cultivars were separated into four clusters during berry development, and their accumulation was regulated by the evolution of the genes related to the LOX-HPL pathway. A correlation study revealed that the expression of the *VvLOXA* gene played an essential role in regulating the accumulation of C6 volatiles in these table grape cultivars.

## 4. Materials and Methods

### 4.1. Chemicals and Standards

The external C6 volatile standards, including hexanal (98.0% purity), (*E*)-2-hexenal (98.0%), 1-hexanol (99.0%), (*E*)-2-hexen-1-ol (96.0%), (*E*)-3-hexen-1-ol (98.0%), (*Z*)-2-hexen-1-ol (96.0%), (*Z*)-3-hexen-1-ol (98.0%), ethyl hexanoate (99.0%), hexyl acetate (99.0%), (*Z*)-3-hexen-1-acetate (98.0%), and hexanoic acid (99.0%), were purchased from Sigma-Aldrich (St. Louis, MO, USA). The internal standard 4-methyl-2-pentanol was also purchased from Sigma-Aldrich, with a purity of 98.0%. Water used in this study was purified from a Milli-Q purification system (Millipore, Bedford, MA, USA). Polyvinylpolypyrrolidone (PVPP) was a product of Sigma-Aldrich. SYBR^®^ Premix Ex TaqTM and Spectrum™ Plant Total RNA Kit were purchased from TaKaRa Bio (Otsu, Shiga, Japan) and Sigma-Aldrich, respectively. A reverse transcription system kit was obtained from Promega (Madison, WI, USA). Other reagents used in this study were purchased from the Beijing Chemical Works (Beijing, China).

### 4.2. Sample Collection

Seven table grape cultivars, including “Xiangfei”, “Moldova”, “Tamina”, “Italia”, “Zaomeiguixiang”, “Muscat of Alexandria”, and “Christmas Rose”, were all cultivated at the experimental vineyard in the Institute of Forestry and Pomology at the Beijing Academy of Agriculture and Forestry Sciences in China (39°58′ N and 116°13′ E). The grapevines of these cultivars were planted in the spring of 2008 and grown in a greenhouse under a two-wire vertical trellis system with 2.5 m row space and 0.75 m plant space. Detailed information on different cultivars is listed in Table 3 and photographs of these cultivars are displayed in Figure S1. Among them, the “Xiangfei”, “Tamina”, “Italia”, “Zaomeiguixiang”, and “Muscat of Alexandria” cultivars exhibited a ‘muscat’ or ‘floral’ character, whereas the “Moldova” and “Christmas Rose” cultivars were classified as neutral varieties. These cultivars appeared to have significantly different berry development duration in the experimental vineyard during the consecutive vintages (2013 and 2014). Grape berries of each cultivar were sampled according to E-L 35 (early veraison, berries begin to color and enlarge), E-L 36 (mid-ripening stage; berries with intermediate Brix values), E-L 37 (end of veraison; berries not quite ripe), and E-L 38 (berries ripe—harvest) of the modified E-L system [44]. At each development stage, about 300 grape berries were randomly collected from three vines in each cultivar. The physicochemical indexes of each cultivar during each sampling interval were immediately determined (Figure S2). Afterwards, the berries were transferred back to our laboratory and then immediately frozen using liquid nitrogen. The frozen samples were stored at −80 °C prior to further analysis.

### 4.3. Extraction of Volatiles

Extraction of volatile compounds from the grape berries followed our previously published method with minor modifications [45]. In brief, after removing seeds and stems, the grape berries (about 100 g) were ground by a stainless grinder (IKA analysis grinder A11) and then mixed with 1 g of polyvinylpolypyrrolidone (PVPP) under liquid nitrogen. The resultant mixture was kept at 4 °C for 4 h, and then centrifuged at 8000 rpm for 15 min at 4 °C to collect the clear juice. Afterwards, the clear juice (5 mL) was mixed with 1 g sodium chloride and 10 µL of 1.00808 g/L 4-methyl-2-pentanol in a 15-mL vial capped with a PTFE-silicon septum. The volatile compounds were extracted using headspace solid-phase micro-extraction (HS-SPME) and then analyzed using Agilent 6890 gas chromatography coupled with Agilent 5975C mass spectrometry (Agilent Technologies Inc., Beijing, China sector). An auto-sampler was operated in SPME mode with an SPME fiber (50/30 µm DVB/Carboxen/PDMS, Supelco, Bellefonte, PA, USA). The sample vial was initially equilibrated at 40 °C for 30 min under agitation, and then the pre-conditioned SPME fiber was inserted into the headspace of the vial to extract volatiles for 30 min at 40 °C under the same agitation conditions. Afterwards, the SPME fiber was immediately inserted into the GC injection port at 250 °C for 8 min to desorb the volatiles. A 60 m × 0.25 mm HP-INNOWAX capillary column with a 0.25 µm film thickness (J&W Scientific, Folsom, CA, USA) was used to separate the volatile compounds under a 1 mL/min flow rate of helium (carrier gas). The oven temperature program was set as follows: 50 °C for 1 min, increased to 220 °C at 3 °C/min, and held at 220 °C for 5 min. The ion source was maintained at 250 °C with the MSD transfer line temperature at 250 °C. A mass scan of *m*/*z* 30–350 was recorded with an ionization voltage of 70 eV.

### 4.4. Identification and Quantitation of Volatiles

For the identification of volatile compounds, C6-C24 n-alkane series (Supelco, Bellefonte, PA, USA) were analyzed using the same chromatographic conditions to calculate the retention indices. The volatile compounds in the grape berries were identified by comparing their retention indices and mass spectrum with their reference standard. The quantitation of volatile compounds was carried out based on the published methods [45]. A synthetic grape berry juice matrix was prepared according to the average sugar and acid level in these grape berries, and was made of distilled water containing 7 g/L tartaric acid and 200 g/L glucose. The synthetic juice matrix was then adjusted to pH 3.3 using 5 M sodium hydroxide solution. All the C6 volatile standards were dissolved in HPLC grade ethanol to yield stock solutions. Each stock solution was mixed in a synthetic matrix to form a standard working solution. The resultant solution was successively diluted to 15 levels. Each standard solution was extracted using the same extraction method as the grape samples and analyzed using the same chromatographic conditions. The calibration curve was established by the peak ratio of the external standard to the internal standard versus the concentration of the external standard. Each C6 volatile compound in these grape berries was quantified using its corresponding external standard.

### 4.5. Total RNA Extraction and Real-Time qPCR Assay

The extraction of the total RNA and the transcript analysis of the key LOX-HPL pathway genes followed a published method [26,27]. Briefly, a mortar was heated at 200 °C for 4 h before the extraction of the total RNA in the grapes to avoid any contamination. Afterwards, after removing seeds and stems, 10 grape berries from each cultivar were ground into a powder using liquid nitrogen in the mortar. The grape powder (100 mg) was used for the total RNA extraction, and extraction was conducted using a Spectrum™ Plant Total RNA Kit (Sigma-Aldrich, Beijing, China sector) according to the manufacturer’s instructions. After the extraction, the quality of the RNA was verified using agarose gel electrophoresis, whereas the concentration of the RNA was determined using the absorbance ratio (A260/A280, 1.8–2.0) on a Nanodrop 2000 spectrophotometer (Thermo Fisher Scientific, Wilmington, DE, USA). Subsequently, the corresponding cDNA was synthesized using the qualified RNA as the template through a Reverse Transcription System Kit (Promega). Quantitation of the relative expression of the LOX-HPL pathway key genes (including *VvLOXs*, *VvHPL1*, *VvADHs*, and *VvAAT*) was carried out under real-time qPCR using the SYBR green method on an ABI7300 Real-Time System (Applied Biosystems, Foster City, CA, USA). The reaction system (10 µL) consisted of 5 µL SYBR Premix Ex TaqII (2×), 0.2 µL ROX Reference Dye (50×) (TaKaRa, Japan), 0.1 µL cDNA (1 µg/µL), 0.2 µL primer mixture (forward primer and reverse primer, 20 µM), and 4.5 µL ddH2O. The real-time PCR was programmed as follows: denaturation at 95 °C for 30 s, followed by 40 cycles of amplification with 94 °C for 10 s and 60 °C for 31 s, and a melt cycle from 60 °C to 95 °C. The primers used in this study are listed in Table S1. The specification of the primer pairs was verified by determining the melt curves and analyzing the size and nucleotide sequence of the PCR product using gel electrophoresis and nucleotide sequencing, respectively. EF1-α (GenBank accession: EC959059), Actin (GenBank accession: EC969944), and UBQ-L40 (GenBank accession: EC929411) were used as internal controls according to previous research [26,27,46]. The difference between the cycle threshold (C_t_) of the target gene and the reference gene (∆C_t_ = C_t Target_ − C_t RefGene_) was used to calculate the normalized expression of the target genes (2^−∆*C*t^) [47]. For each sample (including three independent biological replicates), two independent extraction procedures were performed and three technical replications of real-time qPCR analysis were undertaken. The expression levels of each gene were expressed as a ratio relative to the E-L 35 stage of the “Xiangfei” cultivar, which was set at 1.

### 4.6. Statistical Analysis

One-way and two-factor ANOVA with Duncan’s test at a *p* level of 0.05 were performed using SPSS 20.0 for Windows (SPSS Inc., Chicago, IL, USA). Pearson’s correlation analysis was also carried out using SPSS 20.0 for Windows. The heat maps were obtained using the ‘pheatmap’ package in R (3.1.0). The bar and line graphs were plotted using Origin 8.0 software (OriginLab, Northampton, MA, USA).

## Figures and Tables

**Table 1:** Two-way ANOVA analysis on the concentration of C6 volatiles in seven table grape cultivars during berry development.

Volatiles	Cultivar			Vintage			Cultivar × Vintage		
	F Value	*p*	Sig.	F Value	*p*	Sig.	F Value	*p*	Sig.
Hexanal	75.4	7.49 × 10^−10^	***	468.3	3.68 × 10^−12^	***	64.3	2.19 × 10^−9^	***
(*E*)-2-Hexenal	359.68	1.65 × 10^−14^	***	2215.01	<2 × 10^−16^	***	92.62	1.86 × 10^−10^	***
Total C6 aldehydes	184.85	1.65 × 10^−12^	***	1309.51	3.12 × 10^−15^	***	49.74	1.20 × 10^−8^	***
1-Hexanol	12.81	5.56 × 10^−5^	***	18.41	0.00075	***	2.55	0.06964	
(*E*)-2-Hexen-1-ol	36.56	9.04 × 10^−8^	***	51.22	4.88 × 10^−6^	***	22.64	1.89 × 10^−6^	***
(*Z*)-2-Hexen-1-ol	35.89	1.02 × 10^−7^	***	598.84	6.87 × 10^−13^	***	35.89	1.02 × 10^−7^	***
(*E*)-3-Hexen-1-ol	23.64	1.44 × 10^−6^	***	130.75	1.73 × 10^−8^	***	16.62	1.23 × 10^−5^	***
(*Z*)-3-Hexen-1-ol	707.9	<2 × 10^−16^	***	1833.2	3.02 × 10^−16^	***	293.7	6.72 × 10^−14^	***
Total C6 alcohols	34.15	1.40 × 10^−7^	***	58.07	2.40 × 10^−6^	***	19.84	4.24 × 10^−6^	***
Ethyl hexanoate	2.49	0.07529		23.14	0.00028	***	2.84	0.05024	
Hexyl acetate	17.89	7.94 × 10^−6^	***	1.3	0.274		6.79	0.00155	**
(*Z*)-3-Hexenyl acetate	189.51	1.39 × 10^−12^	***	40.95	1.66 × 10^−5^	***	25.98	8.01 × 10^−7^	***
Total C6 esters	42.34	3.48 × 10^−8^	***	3.3	0.09098		7.24	0.00114	**
Hexanoic acid	11.73	9.11 × 10^−5^	***	78.22	4.18 × 10^−7^	***	4.76	0.00764	**

The F value of the cultivar, vintage, and their interactive effects is calculated using the volatile concentration of C6 compounds in table grapes during the different stages of development. ** and *** indicate a significant effect at *p* < 0.01 and 0.001.

**Table 2:** Pearson’s correlation between C6 volatile compounds and expression of LOX-HPL genes in seven table grape cultivars.

Compound	*AAT*	*ADH1*	*ADH2*	*HPL1*	*LOXA*	*LOXO*
Hexanal	0.478 *		0.525 *	0.533 *	0.701 **	0.529 *
(*E*)-2-Hexenal	0.748 **		0.766 **	0.496 *	0.748 **	
Aldehydes	0.685 **		0.720 **	0.570 **	0.804 **	
1-Hexanol	0.564 **				0.692 **	
(*E*)-2-Hexen-1-ol	0.668 **				0.569 **	
(*Z*)-2-Hexen-1-ol	0.805 **				0.636 **	
(*E*)-3-Hexen-1-ol	0.583 **				0.638 **	
(*Z*)-3-Hexen-1-ol				−0.496 *		
Alcohols	0.655 **				0.568 **	
Ethyl hexanoate				0.452 *		
Hexyl acetate	0.757 **				0.483 *	
(*Z*)-3-Hexenyl acetate						
Esters						
Hexanoic acid	0.772 **		0.564 **	0.509 *	0.664 **	0.613 **
Total amount	0.785 **		0.630 **	0.455 *	0.792 **	

* and ** indicate a significant effect at *p* < 0.05 and 0.01.

**Table 3:** Grape cultivars included in the study.

Cultivar	Color of Berry Skin	Country of Origin of the Variety	Pedigree as Given by Breeder/Bibliography	Breeder	Flavor Description
Xiangfei	Blanc	China	73-7-6 (Muscat Hamburg × Pearl of Csaba) × Cardinal	Institute of Forestry and Pomology	strong muscat and green
Zaomeiguixiang	Rouge	China	Muscat Hamburg × Pearl of Csaba	Institute of Forestry and Pomology	strong muscat
Muscat of Alexandria	Blanc	Greece	Heptakilo × Muscat Blanc a Petits Grains		moderate muscat
Tamina	Rouge	Romania	Bicane × Muscat Hamburg	Gorodea, Gr.; Boian, I.; Lumanare, Zamfiritra	muscat
Italia	Blanc	Italy	Bicane × Muscat Hamburg	Pirovano, Alberto	light muscat, sweet and green
Moldova	Noir	Moldova	Guzal Kara × S.V. 12-375	Zhuravel, M.S.; Gavrilov, I.P.; Borzikova, G.M.; Guzun, N.I.	strong green
Christmas Rose	Rouge	USA	S44-35C × 9-117D	Olmo, Harold, P.; Koyama, Albert T.	light flavor

## Supplementary Materials

Supplementary materials can be found at www.mdpi.com/1422-0067/18/12/2705/s1.

## Abbreviations

Zmgx	Zaomeiguixiang
Alexandria	Muscat of Alexandria
OTV	Odor threshold value
