# Supplementary material for: Differential Expression of VvLOXA Diversifies C6 Volatile Profiles in Some Vitis vinifera Table Grape Cultivars
Source: Int J Mol Sci. 2017 Dec 20;18(12):2705. doi: 10.3390/ijms18122705 (PMC5751306; doi:10.3390/ijms18122705)
Supplement: Supplementary file 1 [file ijms-18-02705-s001.pdf]

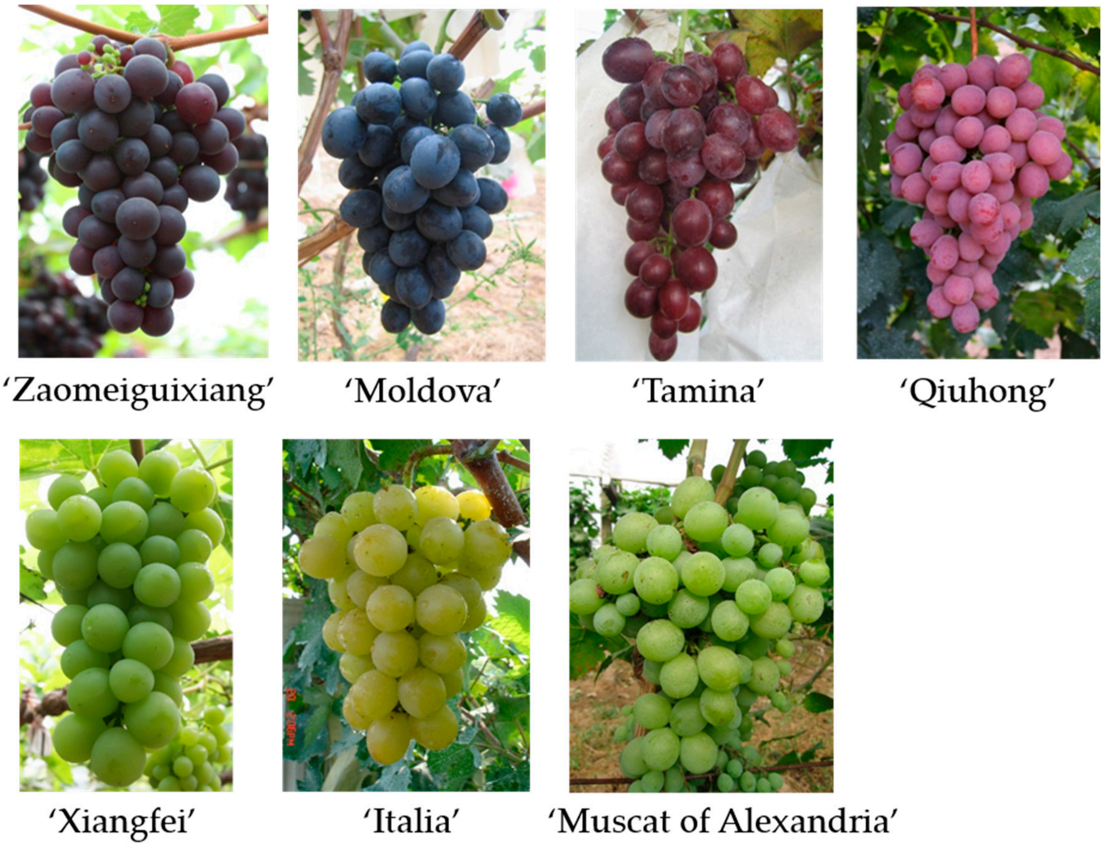

Figure S1. Photographs of seven table grape berries at harvest.

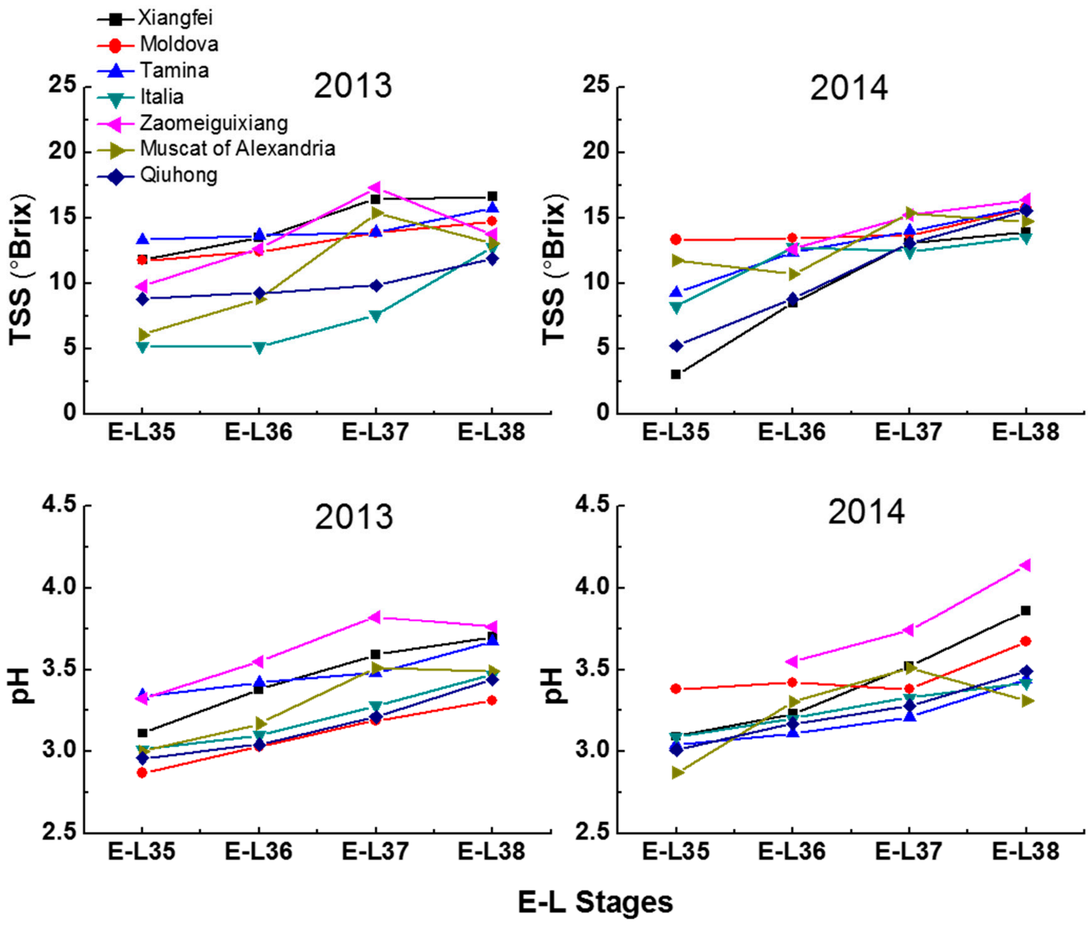

Figure S2. Total soluble solids (TSS) and pH of seven table grape cultivars during berry development in 2013 and 2014.

Table S1. Real-time qPCR primers used in the present study.

Gene	Sense	Antisense	Reference	GenBank accession
EF1-α	GTGCGTCATAGTTTTCTGCCTTCTTCCTTG	CTCAACCAGTTATCTGCCACCGCCTATC	[27]	EC959059
UBQ-L40	CATAACATTTGCGGCAGATCA	TGGTGGTATTATTGAGCCATCCTT	[27]	EC929411
Actin	GCATCCCTCAGCACCTTCCAGCAG	CCACCTCAACACATCTCCATGTCAACC	[27]	EC969944
VvLOXA	GCAAATCAAAGGGACAACGCTGTATGG	TGCTTCCACTGCGGCTTCC	[38]	FJ858255
VvLOXO	TTCCACCCACTCGCCTGATG	GCACCGCACCTGTTTCTTCG	[38]	FJ858257
VvHPL1	AAGTACACCGGCGACATTCGAG	AGCTCTTTACCCTGGCGTGTTG	[27]	HM627632
VvADH1	TCCGTTCTCAGAGATCAACAA	ACTCTCTCATCTCAAGATATTCTATGG	[40]	AF194173
VvADH2	ATTCCAGTCGGCATAAGTGT	TTGCAACTGCATAGACATTGTT	[40]	AF194174
VvAAT	TTAATTCAGGTGACCCGATT	TCTCCATACACATGCCATTAG	[27]	AAW22989
